# Subconjunctival Injection of Mesenchymal Stem Cells for Corneal Wound Healing After Chemical Injury: Impact on Epithelial Coverage, Limbal Ischemia, and Ocular Surface Inflammatory

**DOI:** 10.3389/fmed.2026.1849667

**Published:** 2026-06-30

**Authors:** Shangkun Ou, Yiming Wu, Liying Zhang, Hao Gu, Daoyuan Li, Shiyan Cao

**Affiliations:** 1 Department of Ophthalmology, The Affiliated Hospital of Guizhou Medical University, Guiyang, Guizhou, China; 2 Guizhou Medical University, Guiyang, Guizhou, China; 3 Department of Biomedical Sciences, School of Infection, Inflammation and Immunology, College of Medicine and Health, University of Birmingham, Birmingham, United Kingdom; 4 China Scholarship Council, Beijing, China; 5 Department of Ophthalmology, Guizhou Provincial People’s Hospital, Guiyang, Guizhou, China

**Keywords:** Alkali burn, Cell therapy, Corneal neovascularization, Limbal ischemia, Limbal stem cell deficiency, Ocular surface microenvironment, Ocular surface reconstruction, Regenerative medicine

## Abstract

**Purpose:**

Previous studies in animal models have demonstrated that mesenchymal stem cells (MSCs) are beneficial for reconstructing the ocular surface during corneal wound healing; however, clinical evidence remains scarce. This study aimed to evaluate the clinical safety, feasibility, and preliminary outcomes of subconjunctival umbilical-cord delivered-MSC (UC-MSC) injection as an adjunctive therapy for severe corneal chemical injuries.

**Methods:**

This prospective, single-arm clinical case series enrolled six patients with severe corneal chemical injuries. Human UC-MSCs at passages 5–6 were cultured and verified. A total sample of 4 × 10^6^ cells suspended in 400 μL of 1 × Hanks’ Balanced Salt Solution (HBSS) were subconjunctivally delivered adjacent to the limbus using a 360° circumferential injection technique. Concurrently, all patients underwent amniotic membrane transplantation (AMT) and received a standard 4-week topical regimen (Tobradex, recombinant human epidermal growth factor, and Solcoseryl). Clinical safety, limbal ischemia, inflammatory indices, and epithelial defect areas were assessed through a digital slit-lamp examination.

**Results:**

No localized or systemic adverse events, such as anterior uveitis, progressive ulceration, intraocular infection, or immunological rejection, were observed in any participant. By 4 weeks post-injection, active ocular surface inflammation had significantly decreased in all eyes. Of the five patients with baseline limbal ischemia, four achieved complete revascularization and resolution; the fifth exhibited a marked reduction in ischemia from 100 to 20%. Furthermore, stable corneal re-epithelialization was achieved in five of six eyes, including one with complete paracentral epithelial coverage.

**Conclusion:**

Subconjunctival UC-MSC delivery is a safe and feasible adjunctive therapy for managing severe corneal chemical injuries. This cell-based intervention synergizes with conventional therapies to suppress acute inflammation, rescue limbal perfusion while potentially mitigating subsequent corneal neovascularization, and accelerate the structural reconstruction of the ocular surface microenvironment.

## Introduction

The ocular surface microenvironment (OSM) comprises tissue components, including the cornea, conjunctiva, meibomian glands, lacrimal glands, and the neural network; specific cells, including immune and matrix cells; the extracellular matrix; small molecules; and the microbiome (1–3). In a healthy, non-senescent ocular surface microenvironment, these components function harmoniously to maintain ocular surface homeostasis, corneal transparency, and normal ocular function (4–6). Disruption of the OSM by chemical injury which occurs when a corrosive substance is accidentally introduced to the eye periocular tissues, represents a true ocular emergency (7). In China, chemical injury is a common cause of OSM disruption, predominantly affecting patients aged 15–60 years (8–10). The major pathological changes are inflammation, corneal scarring, limbal stem cell deficiency, complicated glaucoma, and cataract formation (11–13). McCulley divided the clinical course of chemical injuries into four distinct pathophysiological phases: immediate, acute (days 0–7), early repair (days 7–21), and late repair (beyond 21 days) (14, 15). Recovery of the OSM depends on the extent of damage to the corneal epithelium, limbal region, and conjunctiva during the acute phase of chemical injury (16). Based on corneal appearance and the extent of limbal ischemia, Ballen and Roper-Hall established prognostic guidelines for chemical injuries (16, 17). The severity of damage is closely associated with limbal stem cell viability and the degree of inflammatory status. During the acute phase of chemical injury, inadequate anti-inflammatory management and the inability to rescue limbal stem cells frequently contribute to delayed epithelialization, persistent ulceration, corneal perforation, and chronic corneal neovascularization (16, 18). Therefore, anti-inflammatory therapy and preservation of limbal stem cells are critical therapeutic goals for the recovery of OSM during the acute phase of chemical injury. Conventional therapeutic strategies, including topical medications, bandage soft contact lenses, limbal stem cell transplantation, conjunctival transplantation, and amniotic membrane transplantation, remain foundational. However, they often face challenges in fully reconstructing the intricate and multifactorial components of the OSM.

In recent years, mesenchymal stem cells (MSCs), human-induced pluripotent stem cells (hiPSCs)-derived photoreceptor-like cells, and other stem cell-based therapies have been increasingly investigated in ocular regenerative medicine (19–21). Among these, MSCs represent an abundant cell source that can be easily harvested from bone marrow (BM), adipose tissue (AT), and the umbilical cord. Numerous studies have demonstrated that MSCs are characterized by high proliferative capacity (22), robust paracrine activity (23) and the ability to differentiate into mesodermal and non-mesodermal cell lineages (24)all properties that facilitate tissue repair and disease recovery. Most importantly, MSCs exhibit non-immunogenic properties and possess immunomodulatory and immunosuppressive properties (19, 25, 26). Previous studies have shown that using MSCs as an effective approach in the reconstruction of chemically injured could suppress inflammation, improve epithelial recovery, alleviate limbal ischemia, and preserve limbal stem cell function (27, 28). However, because these studies were conducted exclusively in animal models, there is an increasing demand for clinical evidence. To bridge this gap, we report a clinical case series of six consecutive patients with severe corneal chemical injuries treated with subconjunctival human UC-MSC injections. This study aims to evaluate the clinical safety, feasibility, and preliminary therapeutic outcomes of this advanced cell-based adjunctive approach for ocular surface reconstruction.

## Method

### Design

We conducted a non-randomized, single-arm study involving a small cohort of participants. The clinical trial protocol, protocol amendments, and relevant study documentation were reviewed for compliance with Chinese guidelines on clinical research involving human stem cells and were approved by the Institutional Review Board of the Affiliated Hospital of Guizhou Medical University (No. 2023-539). The authors vouch for the accuracy and completeness of the data and analyses, the reporting of adverse events, and the study’s fidelity to the protocol.

### Participants

Six participants (aged 32 to 49 years) with varying baseline severities of corneal chemical injury were enrolled. Although all presented eyes exhibited the classic triad of ocular surface inflammation, limbal ischemia, and corneal epithelial defects, their baseline injury severity and post-injury clinical phases varied among patients, as detailed individually in Table 1.

**Table 1:** Patient characteristics and clinical summary before and after cell injection.

Patient No.	Eye	Disease subtype	Before cell injection			4 Weeks after cell injection		
Sex, and age			Limbal ischemia (%)	Inflammatory index (%)	Size of epithelial defect (%)	Limbal ischemia (%)	Inflammatory index (%)	Size of epithelial defects (%)
1, M, 36 yr	OD	3 days after acid burn	100	77.8	92.6	0	11.1	0
2, M, 42 yr	OS	20 days after alkali burn	42.4	77.8	54.2	0	22.2	0
3, M, 42 yr	OS	20 days after alkali burn	100	66.7	63.6	0	11.1	0
4, M, 44 yr	OD	1 day after dyestuff injury	100	100	100	20	22.2	71.4
5, M, 32 yr	OS	20 days after alkali burn	71.6	66.7	86.3	0	11.1	0
6, M, 49 yr	OD	3 months after alkali burn	—	—	100	—	—	0

### Human umbilical cord MSC culture and quality control

Umbilical cords were obtained during Cesarean section from healthy donors in accordance with the Declaration of Helsinki for research involving human subjects. From the study was approved by the institutional review board of the Affiliated Hospital of Guizhou Medical University, and written informed consent was acquired from all donors. Crucially, all umbilical cord donors underwent comprehensive prenatal care assessments and tested negative via serological screening for major infectious diseases prior to tissue procurement. Isolation of human UC-MSCs was performed as previously described by Fu et al. (29). Briefly, following disinfection via three washes in 1 × PBS containing 2% penicillin–streptomycin, the umbilical cord was cut into approximately 1-cm pieces in length. The mesenchymal tissue (in Wharton’s jelly) was separated from the umbilical arteries, veins, and umbilical cord adventitia.

The isolated Wharton’s jelly was diced into approximately 1 cm^3^ cubes and incubated in a digestion solution containing 2 mg/mL collagenase IV and 5 U/mL hyaluronidase at 37 °C for 4–5 h. Dissociated mesenchymal cells were further dispersed in alpha-modified Eagle’s medium supplemented with 10% fetal bovine serum, 100 U/mL penicillin/streptomycin, and 2 mM L-glutamine and then plated in 100-mm dishes at a density of 3 × 10^5^ cells/mL. These primary mesenchymal cells were either used directly for culture or cryostored in liquid nitrogen for later use. According to the criteria published in Cytotherapy (30), the cultured UC-MSCs were characterized using flow cytometry using cell surface markers, including mesenchymal markers (CD44, CD90, CD105), hemopoietic markers (CD34, CD45), cell adhesion molecules (CD29), endothelial markers (CD31), and MHC class II (HLA-DR). In addition, the multilineage differentiation potential of the UC-MSCs isolated via this standardized protocol has been comprehensively verified and reported in our previous study (31). The detailed flow cytometry protocol is as follows: a cell suspension of MSCs was obtained by trypsinization and centrifugation—all centrifugation steps were performed for 3 min at 1,000 rpm. The cells were then washed in 500 μL of 1 × PBS to remove any remaining culture medium. Following centrifugation, the cell pellets were resuspended in 2% BSA for 20 min on ice or at 4 °C. After centrifugation, the cell pellet was resuspended in 100 μL antibody (5 μL antibody in 100 μL 1% BSA) for 30 min on ice. Finally, the cells were washed twice with 1 × PBS, and the samples were then run through flow cytometry.

### Surgical procedure and follow-up

The surgical procedure was performed under topical anesthesia. A total of 4 × 10^6^ UC-MSCs (passages 5–6), suspended in 400 μL of 1 × Hanks’ Balanced Salt Solution (HBSS), were injected subconjunctivally adjacent to the limbal region. The injection was administered through a single conjunctival entry site using a 1-mL syringe equipped with a specialized ophthalmic injection needle; during injection delivery, the needle was carefully rotated 360° to ensure a uniform circumferential distribution of the 400-μL cell suspension. This dose of 4 × 10^6^ cells was considered clinically safe, as a recent dose-escalation trial demonstrated an excellent safety profile of subconjunctival MSC deliveries of up to 6 × 10^6^ cells split across two injection sites (32). The amniotic membrane was transplanted to the ocular surface and secured with 10–0 absorbable surgical polypropylene sutures. Postoperatively, patients received a topical regimen comprising Tobradex (antibiotic [tobramycin] and corticosteroid [dexamethasone]) eye drops (0.1 mL, t.i.d.), Tobradex eye ointment (0.1 mg, q.n.), recombinant human epidermal growth factor (0.1 mL, t.i.d.), and Solcoseryl eye drops (0.1 mL, q.i.d.). Two to three weeks after transplantation, the amniotic membrane dissolved or was removed.

### Outcome measures

Corneal limbal ischemia, inflammation, and corneal epithelial defects (visualized by sodium fluorescein staining) were clinically evaluated weekly by slit-lamp examination. Following a well-established protocol (33), limbal ischemia and epithelial defect areas were quantified using ImageJ software. These were calculated as the percentage of the affected lesion area/arc relative to the total corneal surface or limbal circumference, respectively. Inflammatory index values were assigned using the same protocol (33), based on three parameters: ciliary hyperemia (absent, 0; present but extending less than 1 mm, 1; hyperemia extending between 1 and 2 mm, 2; and present and extending more than 2 mm, 3); central corneal edema (absent, 0; present with visible iris details, 1; present without visible iris details, 2; present without visible pupil, 3); and peripheral corneal edema (absent, 0; present with visible iris details, 1; present without visible iris details, 2; present with no visible iris, 3). The final inflammatory index was obtained by summing the scores of these parameter and dividing it by 9. To ensure reproducibility and minimize observer bias, all clinical slit-lamp examinations and digital photographs were evaluated independently by two experienced clinical ophthalmologists. Any discrepancies in scoring were resolved through mutual discussion until a consensus was reached.

### Data analysis

Due to the exploration nature and small sample size of this clinical case series, data were analyzed using descriptive statistics. Continuous clinical parameters (percentages of limbal ischemia and epithelial defects) and semi-quantitative inflammatory index values are presented as descriptive trends over time or individual patient metrics.

## Results

### Patient characteristics

Patients hospitalized between 11 March 2020 and 21 July 21 2025 were enrolled. We injected UC-MSCs subconjunctivally into six eyes of 6 participants (age range, 32–49 years) with corneal wounds induced by chemical injuries (Table 1). Of these participants, four suffered alkali burns, one sustained an acid burn, and one was injured by a chemical dyestuff. Of the six patients, two were at the acute stage (during 0–3 days after the injury), three were at the early recovery stage (during 7–21 days after the injury), and one was at the late recovery stage (late 20 days after the injury). At baseline, all patients exhibited limbal ischemia, epithelial defects, and ocular surface inflammation.

### Features of the cultured UC-MSCs

**Figure 1:**
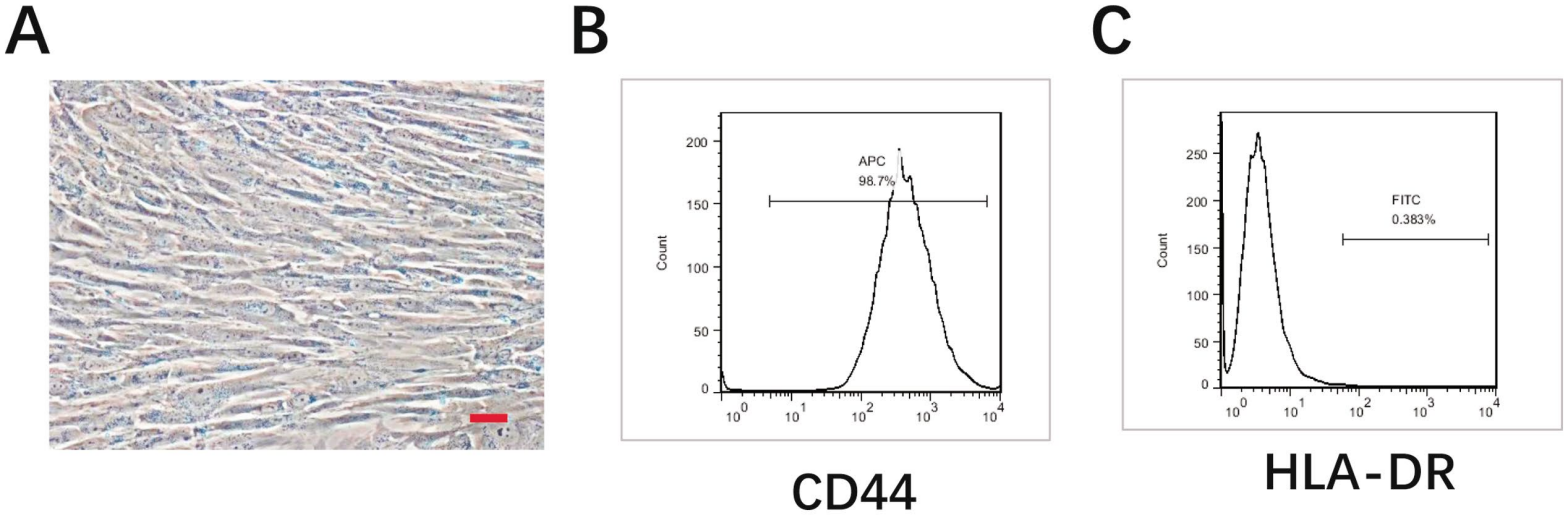
Characterization of MSCs. **(A)** The morphology of MSCs. MSCs were spindle-shaped (bar = 50 μm). **(B)** Flow cytometry analysis of mesenchymal stem cells (MSCs). Expression of CD44 in isolated MSCs. MSCs did not express HLA-DR.

Cultured UC-MSCs at passages 5–6 were utilized for all interventions. To meet institutional safety compliance standards established by the special committee, etiological screening was performed at passage 3, and MSC characteristics were evaluated across subsequent passages. Under optical microscopy, cultured UC-MSCs exhibited a typical homogeneous spindle-shaped morphology (Figure 1A). Flow cytometry analysis confirmed high expression of key mesenchymal surface cell markers, such as CD44 (98.7%) (Figure 1B, anti-human CD44, Molecular Probes, A14749), and negative expression of the immunogenic/hematopoietic marker HLA-DR (0.383%) (Figure 1C, Anti-human HLA-DR, Invitrogen, MHLDR01). In addition, the cells maintained high expression positivity (>95%) for CD29, CD90, and CD105, while remaining negative for CD31, CD34, and CD45 (data not shown).

### Effects of UC-MSCs on limbal ischemia recovery

**Figure 2:**
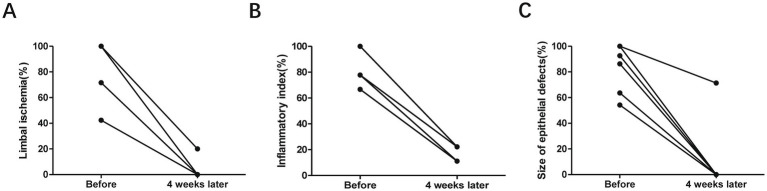
Corneal limbal ischemia, inflammatory index, and size of epithelial defects. 4 weeks after MSCs injection, the injury limbal ischemia **(A)** was decreased; the inflammatory index was decreased **(B)**; and the size of epithelial defect was decreased **(C)**.

**Figure 3:**
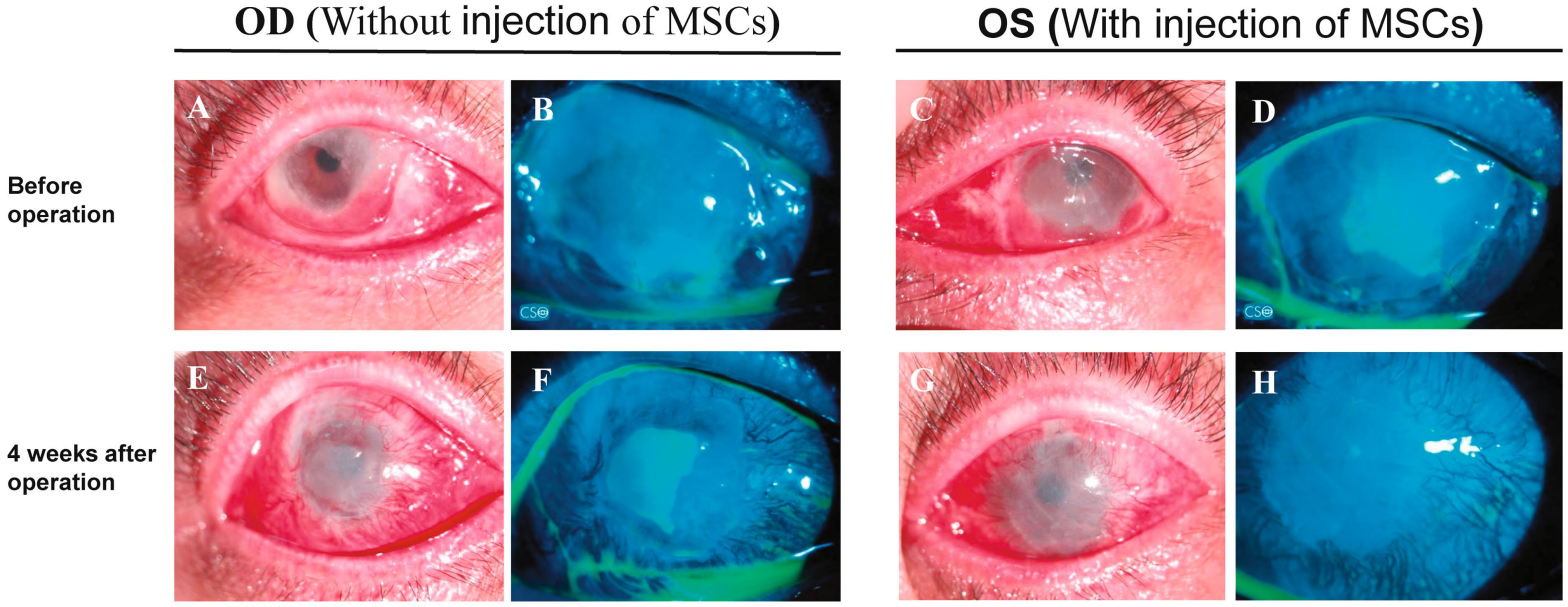
Comparison of the therapeutic effect with and without injection of MSCs. **(A–D)** Representative images of before operation. Without injection of MSCs **(A,B)** and with injection of MSCs **(C,D)**. **(E–H)** Representative images of 4 weeks after operation. Without injection of MSCs **(E,F)** and with injection of MSCs **(G,H)**.

Limbal ischemia is a critical prognostic indicator for ocular surface burns. It can lead to a series of pathologies, including LSCD, persistent epithelial defects, and even corneal perforation. In this study, five of the six participants exhibited limbal ischemia at baseline. Patient 6 was excluded from this analysis due to pre-existing corneal neovascularization. By 4 weeks of injection, limbal ischemia had decreased across all affected eyes. Except for the limbal ischemia of patient 4, which decreased to 20% from 100%, the limbal ischemia of the remaining patients completely disappeared (Table 1; Figure 2A). Notably, patient 5 was injured in both eyes by alkali burns. Although both eyes were treated with a conventional therapeutic method, the range of limbal ischemia was substantially more extensive in the left eye than in the right eye. However, 4 weeks after cell injection, the limbal ischemia in the left eye treated with UC-MSC had recovered to a large extent, whereas the untreated right eye continued to show signs of limbal ischemia (Figure 3).

### Anti-inflammatory effects of UC-MSCs in the cornea

Slit-lamp examination revealed severe baseline inflammation in all treated eyes, which was visibly reduced by 4 weeks after UC-MSC treatment (Figure 2). We used the inflammatory index to assess the anti-inflammatory effects of UC-MSCs in the cornea. At 4 weeks postoperatively, the inflammatory index had decreased in all treated eyes (Table 1; Figure 2B). Notably, in the patients with bilateral burns (Patient 5), baseline ciliary hyperemia, central corneal edema, and peripheral corneal edema were initially more severe in the left eye than in the right eye at baseline. However, at 4 weeks post-intervention, the inflammatory state was better in the UC-MSC-treated left eye than in the conventionally treated right eye (Table 1; Figure 3).

### Effects of UC-MSCs on epithelial recovery

Corneal epithelial staining with fluorescein was used to evaluate epithelial defects. All corneal epithelium defects were >50% at baseline (before cell injection); in patients 4 and 6, the entire corneal epithelium was absent. By 4 weeks post-injection, the corneal epithelium had healed in five of the six eyes. Although patient 4 presented with severe limbal ischemia and inflammation, the paracentral cornea was successfully covered by epithelium (Table 1; Figure 2C). In the bilaterally injured patient 5, at baseline, baseline epithelium damage was more severe in the left eye than in the right eye. At 4 weeks after UC-MSC injection, the corneal epithelium completely recovered in the UC-MSC-treated eye, whereas the conventionally treated right eye displayed fluorescent staining consistent with a persistent epithelial defect (Figure 3).

### Safety

We observed no cases of anterior uveitis, progressive ulceration, intraocular infection, or immunological rejection in any patient following UC-MSC injections. Furthermore, no abnormal general health findings were observed during the follow-up period.

## Discussion

Our findings demonstrate that the subconjunctival injection of human UC-MSCs in patients with chemical injury-induced corneal wounds may facilitate the reconstruction of the OSM, as evidenced by improvements such as limbal ischemia, inflammation, and re-epithelization, and leading to better prognosis, leading to improved clinical improvements. Despite the limitations of this small, single-arm study involving six patients who were followed for 4 weeks, the procedure was demonstrated to be safe.

OSM disruption caused by chemical injury is an ophthalmological emergency with a high risk of blindness. Because these injuries often result in extensive damage, severe inflammation, and permanent disruption of the OSM, immediate assessment and initiation of therapeutic interventions are crucial to restore the OSM (34). Although conventional treatment modalities are indispensable, they often have the limited ability to fully reconstruct the highly complex and severely damaged OSM. As the injury progresses, the corneal epithelium may be lost, and the limbal area, which is the location of the limbal stem cells, may be destroyed by the chemical substance and inflammation (18). As the disease progresses and tissue damage worsens, corneal ulceration may eventually occur (35–37). Recently, MSCs have emerged as a promising therapeutic approach for the treatment of corneal chemical burns (38–40).

There are four primary methods for MSC delivery: transplantation of an MSC-seeded amniotic membrane (41), subconjunctival MSC injection (42–44), intrastromal implantation of MSCs into surgically created pockets surrounding the injury using a 15° knife, and topical application of eye drops to the corneal surface (45). Based on our previous study, we selected a subconjunctival injection around the cornea as the route for administering MSCs. The cell density was 1 × 10^6^ cells per 0.1 mL, yielding a total cell dose of 4 × 10^6^. For most patients, a single MSC injection proved sufficient for therapy. However, patient 4 still exhibited persistent limbal ischemia, inflammation, and corneal epithelial defects at 4 weeks post-injection. Regarding cell retention, Yao et al. found that numerous DiI-labeled MSCs had accumulated in the subconjunctival tissue for at least 7 days post-injection (42).

Severe chemical injuries are ocular emergencies that induce continuous, progressive tissue damage. The earlier the injury is assessed and appropriate therapeutic approaches are initiated, the better the prognosis is likely to be (15). However, preparation of UC-MSCs take 3–4 days for thawing and passage cells. Infiltration of neutrophils and macrophages, along with the release of inflammatory mediators, occurs early during corneal wound healing, typically 12–24 h after the initial injury (46). Measures to shorten the preparation time warrant further investigation. Currently, MSCs can be obtained from a variety of tissue types, including bone marrow (BM) (47), adipose tissue (AT) (48), and the umbilical cord (UC) (49). In comparison with BM-MSCs and AT-MSCs, UC-MSCs are safer, more accessible, and more cost-effective. Recent studies have provided novel insights into the beneficial role of UC-MSCs in the regeneration of corneal epithelium (50, 51). The UC-MSCs are an ideal cell source for application in the ocular surface.

Furthermore, MSCs are well-known to possess inhibitory effects on inflammation. To date, numerous studies have demonstrated that MSCs inhibit inflammatory cell infiltration and reduce pro-inflammatory cytokine production. Specifically, MSCs have been shown to significantly reduce CD68^+^ and CD4^+^ cellular infiltration and to downregulate macrophage inflammatory protein-1 alpha (MIP-1a), IL-2, IFN-*γ*, TNF-a and VEGF (42, 43, 45, 52, 53). Our results corroborate these findings, demonstrating that the subconjunctival injection of MSC significantly attenuated the inflammation in the locally burned cornea. The inflammatory index in all patients dramatically decreased. Notably, in Patient 5, transplantation of MSCs achieved a better outcome in suppressing corneal inflammation (Table 1; Figure 3).

Ischemia frequently accompanies corneal chemical injuries. The prognosis of corneal wound induced by chemical injury is influenced by the ischemia of the limbal area; the wider and deeper the ischemic area of the limbus, the worse the prognosis (16, 17, 54). The corneal epithelium originates from limbal epithelium stem cells (LESCs). LESCs reside in a unique microenvironment known as the LESC niche (LESCN) within the limbal basal layer. The LESCN is composed of epithelium, stroma cells, mesenchymal cells, nerve, and blood vessels (55–57). Chemical injuries destroy the LESCN, including its resident cells, extracellular matrix, and supporting vasculature, ultimately leading to blood vessels. It is well known that the destroyed blood vessels cause ischemia. Destruction of the limbus is driven by both direct chemical diffusion and the subsequent invasion of the inflammatory response. Substantial evidence supports the findings that MSCs produce a variety of pro-angiogenic paracrine factors and may differentiate into endothelial-like cells (58); consequently, MSC-based transplantation has been widely used in ischemia therapy in other tissues, such as the limbs (59) and heart (60). Although the exact mechanisms of MSCs in treating limbal ischemia remain to be fully elucidated, a subconjunctival injection of MSCs yielded significantly greater improvements in limbal ischemia following corneal chemical wounds (Table 1; Figure 2). Thus, MSC transplantation likely achieves a better prognosis by promoting revascularization, which, in turn, reconstructs the LESCN and protects the LSCs.

There are competing hypotheses about how MSCs restore the OSM. Ye (61) and Jiang (62) demonstrated that MSCs can transdifferentiate into corneal epithelial cells to enhance wound healing and improve reconstruction of the corneal surface. Conversely, other researchers have argued that the beneficial effects of MSCs primarily stem from inhibiting inflammation and modulating the OSM (42, 43, 52, 53). We found that the subconjunctival injection of MSCs accelerated corneal epithelial recovery. For example, patient 6 presented with a central corneal epithelial defect that had persisted for 3 months despite conventional therapy, with the risk of corneal perforation. However, the corneal epithelium recovered successfully after MSC transplantation (Figure 2). Moreover, in patient 3, the recovered corneal epithelium was better after MSC transplantation than without it (Table 1; Figures 2, 3). Regardless of the effects of MSCs in corneal wounds induced by chemical injury, MSCs may transdifferentiate into corneal epithelial cells or inhibit inflammation. Our findings suggest that subconjunctival injections are a good alternative for the clinical application of MSCs to corneal chemical injury. Taken together, the accelerated epithelial healing observed in our study is closely associated with the potent anti-inflammatory environment modulated by UC-MSCs. This finding is supported by the significant decline in the postoperative inflammatory index across all treated eyes. Patient 3 demonstrated a notably superior resolution of corneal inflammation in the MSC-treated eye compared to the untreated contralateral eye (Table 1; Figure 3).

Nevertheless, several intrinsic limitations of this study should be acknowledged. First, this trial utilized a prospective, single-arm design with a small sample size and lacked a standardized parallel control group. Because severe chemical burns represent acute, sight-threatening emergencies, our medical team opted for a comprehensive, intensive therapeutic regimen to prioritize visual salvage and accelerate ocular surface stabilization. Consequently, completely isolating the precise independent therapeutic contribution of UC-MSCs from the potent anti-inflammatory effects of AMT and the epithelial-migratory benefits of recombinant human epidermal growth factor and Solcoseryl remains a scientific challenge. However, the intra-patient contralateral comparison in patient 5, where both eyes received identical adjuvant treatments but only one received UC-MSCs, yielded a significantly superior and accelerated outcome in the MSC-treated eye. This critical clinical observation strongly suggests that UC-MSCs may exert powerful additive and synergistic effects extending beyond the capabilities of conventional aggressive therapies alone. Future randomized, controlled trials with segmented treatment arms are required to fully delineate these independent variables. Second, the current study is constrained by a relatively short-term observation period of only 4 weeks and a lack of granular, high-frequency longitudinal assessment intervals. This tracking gap was primarily due to a practical clinical limitation of our multi-modal surgical design: The co-transplant amniotic membrane served as a dense biological patch that completely covered the cornea, physically obscuring direct slit-lamp visualization and precise sodium fluorescein staining scores of the underlying epithelium during the first 2–3 postoperative weeks. Consequently, determining the exact temporal onset of cellular action, as well as the long-term durability of US-MSC therapeutic effects, remains challenging. Acknowledging this short-term follow-up is critical, as extended longitudinal monitoring will be essential in future trials to accurately characterize cell decline kinetics, evaluate their long-term efficacy against corneal neovascularization caused by diseases or during treatment potentially, and establish optimal dosing intervals for potential repeat administrations. Finally, although the basic morphology and surface markers of the UC-MSCs were verified, their full tri-lineage differentiation potential and long-term cell viability dynamics were not comprehensively assessed in this study. This limitation highlights the need for standardization and validation in larger clinical trials.

In summary, our study demonstrates that the subconjunctival injection of UC-MSCs suppresses inflammation, resolves limbal ischemia, and accelerates corneal re-epithelialization in patients with chemically induced corneal injury, with no observed adverse effects. Our findings suggest that the use of UC-MSCs may represent an effective, safe, and viable adjunctive treatment for the clinical management of corneal chemical burns.

## Data Availability

The original contributions presented in the study are included in the article/supplementary material, further inquiries can be directed to the corresponding authors.
